# WHO HEARTS programs in high-income countries: Implementation, outcomes, and implications for global hypertension control

**DOI:** 10.1016/j.ajpc.2026.101579

**Published:** 2026-03-25

**Authors:** Jiancheng Ye, Sophie Bronstein, Haoxin Chen

**Affiliations:** a Weill Cornell Medicine, Cornell University, New York, NY, United States; b Feinberg School of Medicine, Northwestern University, Chicago, IL, United States

**Keywords:** Hypertension, WHO HEARTS, High-income countries, Primary health care, Health systems strengthening, Cardiovascular disease prevention, Evidence-based protocols, Team-based care, Quality improvement, Public health policy

## Abstract

Despite advanced healthcare systems and near-universal coverage, hypertension control rates have stagnated in many high-income countries (HICs), even as this condition remains the leading preventable cause of cardiovascular disease and premature mortality worldwide. The WHO HEARTS technical package, initially designed for low- and middle-income countries (LMICs), offers a comprehensive framework for strengthening hypertension management through six core components: healthy lifestyle counseling, evidence-based protocols, access to essential medicines and technology, risk-based cardiovascular disease management, team-based care, and systems for monitoring. Modeled on prior global analyses of WHO HEARTS in LMICs, this structured program report synthesizes available evidence on HEARTS implementation and outcomes in HICs, drawing on national registry data, PAHO/WHO program reports, and pooled epidemiological surveys. Results demonstrate that HEARTS is both feasible and effective in high-resource settings, with early adopters achieving substantial improvements in hypertension control. This report also identifies key barriers to HEARTS implementation in HICs, including therapeutic inertia, physician resistance to standardized protocols, fragmented electronic health record systems, and persistent socioeconomic inequities. However, HICs possess distinct advantages including advanced monitoring infrastructure, trained multidisciplinary teams, reliable medication supply chains, and financing capacity. These findings demonstrate that HEARTS provides a relevant and necessary framework for HICs to overcome systemic barriers, close equity gaps, and achieve sustained improvements in hypertension control. Expanding HEARTS across high-resource settings represents a critical opportunity to reverse declining control trends and prevent avoidable cardiovascular disease globally.

## Graphical abstract

**Figure.** Global Implementation of HEARTS: HICs, LMICs, Reference Models, and Benchmark Countries.

**Figure:**
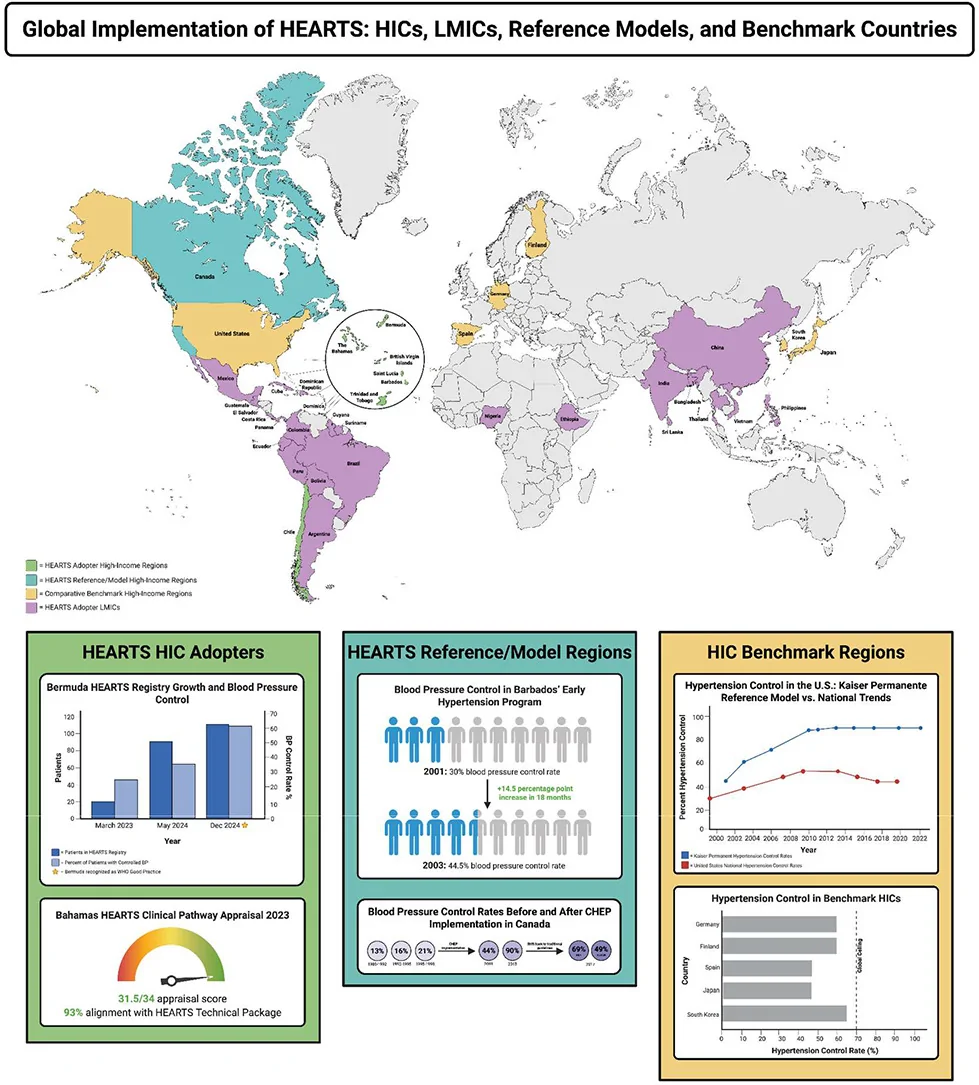

## 1 Introduction

The global burden of hypertension has doubled since 1990, rising from 650 million to 1.3 billion adults aged 30–79 years, with two-thirds now residing in low- and middle-income countries (LMICs) [1]. Yet only one in five adults worldwide have their blood pressure (BP) adequately controlled [2]. Although the burden is greatest in LMICs, high-income countries (HICs) continue to face substantial challenges. Globally, as of 2023, only 54% of individuals with hypertension are diagnosed, 42% receive treatment, and 21% achieve control [1]. In HICs, approximately 28% of adults with hypertension have controlled BP, with considerable variation between countries. Control rates range from below 30% in Japan and Finland to approximately 60% in Canada and South Korea [3]. In the United States, 51% of adults with hypertension were on medication in 2021–2023, but only 21% had BP <130/80 mmHg [4]. Japan reported treatment rates around 47% with control rates below 30% in 2019, and Finland similarly showed treatment levels below 50% and control rates below 30% [3].

Hypertension in HICs is further complicated by multimorbidity, particularly diabetes, obesity, and chronic kidney disease (CKD), which increases both clinical complexity and healthcare costs [1,5]. Socioeconomic inequities also persist: lower income, lower educational attainment, and social isolation are all associated with reduced awareness, treatment, and control, even in settings with universal health coverage [6]. Some HICs, including the United States, have experienced stagnation or declines in national control rates in recent years [7].

The WHO HEARTS technical package offers a structured framework to address these challenges through standardized treatment protocols, reliable access to medicines and validated devices, risk-based cardiovascular prevention, team-based care, and robust monitoring systems [8]. Although primarily scaled in LMICs, the package was informed by high-income models such as the Canadian Hypertension Education Program (CHEP) and Kaiser Permanente's hypertension registry [9]. Kaiser Permanente's registry-based program has exceeded the global ceiling of 70% control, beginning at 44% in 2000 and reaching 90% in Northern California by 2013 [10]. Since then, Kaiser Permanente has focused on maintaining these control levels, reducing disparities, and integrating hypertension management into broader cardiovascular care. Canada's CHEP demonstrated increased hypertension control rates after its launch in 1999, rising from under 15% in the early 1990s to approximately 65–70% in the late 2000s. However, these levels plateaued as the program's priorities shifted away from systems-level continuous monitoring toward more traditional guidelines [11]. These reference models demonstrate the importance of sustaining HEARTS-style approaches even in HICs, as their implementation has exceeded national BP control levels while discontinuation has been associated with declining control.

More recently, Bermuda became the first HIC to formally adopt HEARTS. By 2024, BP control rates at the Hamilton Health Centre hypertension registry had increased from 25% to 62%. The program was recognized by the Pan American Health Organization/World Health Organization (PAHO/WHO) as a Non-Communicable Disease Good Practice for its emphasis on equity, task-sharing, and continuous quality improvement [12,13]. Other high-income jurisdictions in the Caribbean, including Barbados and the Bahamas, have also joined the HEARTS Initiative through PAHO, further demonstrating its feasibility in HIC contexts [14]. This structured program report aims to examine: (1) the feasibility and outcomes of WHO HEARTS implementation in HICs; (2) the key facilitators and barriers that distinguish HIC from LMIC implementation; and (3) how HEARTS components can be adapted to high-resource contexts to overcome persistent inequities and reverse stagnating control trends.

To date, a comprehensive evaluation of HEARTS implementation specifically in HICs has not been published. A landmark analysis by Moran et al. documented HEARTS implementation and outcomes across 32 LMICs, identifying key drivers of success including standardized protocols, registry-based monitoring, and task-sharing [15]. The present report applies an equivalent structured framework to HIC settings, providing the complementary high-income country analysis

## 2 Methodology

The WHO HEARTS technical package is a standardized framework for strengthening hypertension control through primary health care. Its six components—Healthy lifestyle counseling, Evidence-based protocols, Access to essential medicines and technology, Risk-based CVD management, Team-based care, and Systems for monitoring—provide a structured approach that has been applied widely in LMICs but is increasingly relevant to HICs [11]. In HICs, hypertension prevalence remains high, control rates have stagnated, and multimorbidity and socioeconomic inequities complicate management [1,5,7]. The following subsections describe how each HEARTS module can be applied and adapted in HIC contexts, highlighting countries that have successfully implemented HEARTS principles.

### 2.1 Study design

This is a structured program report examining the implementation and outcomes of the WHO HEARTS technical package in HICs. The design mirrors that of Moran AE study, which used the same approach to document HEARTS implementation across 32 LMICs [15]. We synthesized data from four source categories: (1) peer-reviewed publications identified through a search of PubMed/MEDLINE using the terms "WHO HEARTS," "HEARTS technical package," "hypertension control program," and country-specific terms for HIC adopters (Bermuda, Bahamas, Barbados, Chile, Trinidad and Tobago, British Virgin Islands); (2) PAHO/WHO program reports, policy briefs, and NCD Good Practice documentation; (3) the HEARTS Compendium of Essential Clinical Tools and HEARTS 2.0 documentation; and (4) pooled national survey data from the NCD Risk Factor Collaboration (NCD-RisC) for longitudinal trends in HIC hypertension awareness, treatment, and control.

Countries classified as high-income by the World Bank were eligible for inclusion as HEARTS adopters. Countries classified as low- or middle-income — including Cuba and Saint Lucia — were excluded from the primary analysis of HEARTS HIC adopters, despite appearing in PAHO regional documents, though their data are referenced for comparative context where relevant. CHEP (Canada) and Kaiser Permanente Northern California (United States) were included as pre-HEARTS reference programs that informed the design of WHO HEARTS and serve as high-resource implementation benchmarks.

The primary objective of this report is to determine whether the WHO HEARTS technical package is feasible and associated with measurable improvements in hypertension control when implemented in high-income country settings. This builds directly on the LMIC analysis of Moran et al., which established the effectiveness of HEARTS in lower-resource contexts and identified implementation drivers; our report asks whether those findings translate to high-resource settings with different health system structures, guideline thresholds, and baseline infrastructure.

Our central hypothesis is that WHO HEARTS implementation in HICs is both feasible and associated with improvements in hypertension coverage and control, and that HICs adopting HEARTS-consistent frameworks achieve superior outcomes compared to non-adopting HICs of comparable resource levels.

Consistent with the monitoring framework used in the Moran et al. LMIC analysis and the HEARTS Compendium of Essential Clinical Tools, the following standard metrics were used to evaluate program outcomes where reported:

Primary metrics:

• Proportion of enrolled hypertensive patients achieving BP control (<140/90 mmHg per WHO HEARTS standard, with notation where country-specific thresholds differ — see E: Evidence-Based Protocols section and Data Collection and Analysis)
• Registry enrollment as a proportion of the estimated hypertensive catchment population (coverage rate)
• Change in BP control rate from baseline to most recent reporting period

Secondary metrics:

• Treatment coverage (proportion of hypertensive patients prescribed antihypertensive medication)
• Proportion of enrolled patients lost to follow-up or missing visits in the prior 3 or 12 months
• Facility-level adoption rate (number of PHC centers implementing HEARTS protocols over time)
• Compliance score on the HEARTS pathway appraisal checklist (where administered)
• Comparative trends in hypertension control for non-HEARTS HIC benchmark countries from NCD-RisC pooled data

#### 2.1.1 H: healthy lifestyle counseling

Population-level interventions to reduce salt intake, improve diet, and encourage physical activity have been fundamental to hypertension prevention in HICs. National campaigns such as the UK's salt-reduction program, U.S. dietary guidelines, and Japan's nutritionist-led salt restriction programs demonstrate how policy can lower BP across populations [1]. However, obesity, physical inactivity, and high sodium consumption remain widespread. The WHO Global Report on Hypertension highlighted that excessive salt intake contributed to two million cardiovascular deaths in 2019, and average daily intake in some HICs is three times the recommended level [1]. Lifestyle behaviors are strongly influenced by socioeconomic factors such as low income, educational attainment, and social isolation, and vulnerable groups have lower rates of adherence to dietary guidelines and physical activity [6]. Lifestyle change programs in HICs increasingly integrate digital health technologies such as mobile applications, wearable sensors, and telehealth behavioral counseling, providing new possibilities for personalized risk reduction [7].

#### 2.1.2 E: evidence-based protocols

Streamlined algorithmic treatment protocols are central to HEARTS. National and local practice guidelines in HICs, such as the United States' 2017 ACC/AHA guidelines (≥130/80 mmHg threshold) and the European Society of Hypertension (≥140/90 mmHg), illustrate how evidence is translated differently across settings [9,16].

This divergence in BP thresholds has direct implications for HEARTS implementation and outcome measurement in HICs. The WHO HEARTS technical package defines BP control as <140/90 mmHg — consistent with European Society of Hypertension and most LMIC guidelines — but this threshold does not align with the US ACC/AHA guideline target of <130/80 mmHg [9,16]. As a result, reported HEARTS control rates in Caribbean HIC adopters (using <140/90 mmHg) are not directly comparable to US national control statistics reported at the stricter threshold. In the US context, a patient considered 'controlled' under the HEARTS standard may still be classified as uncontrolled by national guidelines. When HEARTS is implemented or evaluated in HICs with more stringent national targets, the program's simplified treatment algorithms and BP control definitions should be explicitly adapted to align with local standards. The Moran et al. analysis of LMIC HEARTS programs similarly used <140/90 mmHg as the standard outcome metric; HIC implementations using this threshold should note the limitation when reporting to, or comparing against, countries applying stricter criteria.

However, simplified protocol adoption faces challenges in HICs, including therapeutic inertia, "guideline fatigue," and variations between professional societies [16]. Standardization remains difficult in fragmented systems where multiple guidelines coexist, as in the U.S. In contrast, CHEP and Kaiser Permanente's hypertension registry have demonstrated that streamlined protocols coupled with ongoing tracking and physician feedback can achieve high BP control rates [9]. Kaiser Permanente Northern California was included as a reference program because it remains the only high-income health system to exceed the global control ceiling of 70%, reaching >80% through registry-based management [10]. The Canadian CHEP program was included as another reference, having raised control from 21% in 1999 to approximately 66% by 2006 before rates declined after priorities shifted in 2011 [11].

#### 2.1.3 A: access to essential medicines and technology

Most HICs have universal coverage or insurance providing broad access to antihypertensive medications, and 94% of communities in HICs report access to the four major classes of BP-lowering drugs [7]. However, affordability remains an issue in certain contexts, particularly the United States, where uninsured or underinsured patients face high out-of-pocket costs [6]. Even where medications are available, polypharmacy, poor medication adherence, and inconsistent drug prescribing practices impact effectiveness [5]. In HICs, universal access to validated BP measurement devices is converging with rising interest in home BP monitoring and telehealth-enabled medication titration. Data suggest that home monitoring coupled with healthcare professional feedback translates to higher control rates compared to office-based measurement alone [1,7]. Therefore, digital health tools and networked devices become valuable components for HIC HEARTS adaptation.

#### 2.1.4 R: risk-based CVD management

Risk stratification is a critical component of hypertension management in HICs, where multimorbidity is common. Patients often present with hypertension alongside diabetes, chronic kidney disease (CKD), or hyperlipidemia, which increases both clinical complexity and costs [5]. Integrating risk-based care ensures treatment intensity aligns with total cardiovascular risk rather than BP alone. HEARTS 2.0 emphasizes convergence of hypertension within the interrelated systems of cardiovascular, renal, and metabolic diseases, a model especially relevant to HICs [17]. For example, Bermuda's national HEARTS program utilizes primary care registries to identify high-risk patients requiring intensive control, while Canada continues to use risk calculators and laboratory testing to guide therapeutic decisions [11,12]. This integrated approach not only improves clinical efficacy but also optimizes resource utilization in health systems burdened by multimorbidity.

#### 2.1.5 T: team-based care

In HICs, team-based care has become increasingly institutionalized, with physicians, nurse practitioners, pharmacists, and community health workers collaborating to manage hypertension. Evidence from Canada and the United States indicates that pharmacist-led interventions can enhance control rates by as much as 20% [11,12]. Meanwhile, the UK's National Health Service has expanded its scope to include nurse- and pharmacist-led clinics within primary care networks, seamlessly integrating hypertension management into routine practice [18]. HEARTS extends these models by formalizing task-sharing and making community outreach central. Bermuda and the Bahamas have demonstrated how HEARTS protocols can be implemented across primary-level multidisciplinary teams and yield improvements in both adherence and outcomes [12,19]. Telemedicine further extends team-based models in HICs, facilitating remote monitoring and timely therapeutic adjustments [7].

#### 2.1.6 S: systems for monitoring

Robust monitoring is one of HIC health systems' greatest strengths. Quality registries, electronic health records (EHRs), and national health surveys yield near-real-time data on hypertension diagnosis, treatment, and control. Analysis spanning several decades across 12 HICs demonstrates substantial improvements since the 1980s but stagnating control rates in recent years, rarely exceeding 70% even in leading countries [9]. Canada, South Korea, Germany, and the United States have the highest levels of awareness and control, while Japan, Spain, and Finland lag behind [5,9].

The principles of HEARTS monitoring—registries, dashboards, and feedback loops—are already embedded in many health information systems, but they require reinforcement to maintain momentum. CHEP links performance indicators to clinical practice, while Kaiser Permanente's registry provides ongoing audit-and-feedback cycles [9,11]. Bermuda's HEARTS program illustrates how registry expansion can be rapidly scaled in small HICs, growing from 26 to 85 enrolled patients within one year [12]. Despite these successes, recent stagnation highlights the need for renewed investment in monitoring and accountability systems to reach the World Hypertension League's 80 × 80 × 80 targets [7].

### 2.2 Data collection and analysis

Both CHEP [11] and the Kaiser Permanente Northern California hypertension registry [10] have gained global recognition as models of protocol-based care, standardized treatment, and registry feedback, and were therefore included as reference cases. PAHO reports and policy briefs were reviewed to capture implementation of HEARTS in high-income jurisdictions within the Americas, including Bermuda [12,20], the Bahamas [17], and Barbados [21]. Recent PAHO/WHO reporting provided updated quantitative outcomes for Bermuda through December 2024, including registry size, BP control, and recognition as a WHO Good Practice. This designation signifies that the program was internationally validated as a model from which other countries can learn [13]. National clinical pathway review reports [17] and the HEARTS Compendium of Essential Clinical Tools were reviewed to capture elements of protocol content, implementation quality, and compliance with key drivers of HEARTS [22].

Information was extracted from these sources regarding year of adoption or initiation, number and type of health facilities involved, population coverage where available, level of implementation, and results related to treatment and BP control. For settings with functioning registries, such as Bermuda [12] and Kaiser Permanente [10], information related to patient enrollment and follow-up was extracted to identify trends in control. For situations with reports at the polyclinic or national program level, such as Barbados [21] and the Bahamas [19], data on protocol compliance, population coverage, and implementation fidelity were extracted. Consistent with the HEARTS monitoring framework, particular attention was given to three standard indicators—the number of patients with hypertension enrolled from the catchment population, the proportion of those enrolled who achieved BP control (<140/90 mmHg), and the proportion who were lost to follow-up or missed visits during the last 3 or 12 months. These metrics were abstracted where reported and used to facilitate comparisons across settings.

All BP control outcomes in this report are presented using the WHO HEARTS standard threshold of <140/90 mmHg, consistent with the approach used in the foundational LMIC analysis by Moran et al. [REF] and with the HEARTS Compendium of Essential Clinical Tools [21]. In HICs where national guidelines specify a more stringent threshold — notably the United States, where the 2017 ACC/AHA guidelines define control as <130/80 mmHg — this distinction is noted explicitly in the relevant results. Readers should interpret cross-country comparisons with this threshold difference in mind, as applying different definitions to the same patient population can yield substantially different reported control rates.

Comparative data were also integrated to contextualize the broader performance of hypertension control in HICs. Pooled national surveys compiled by NCD-RisC were reviewed to describe long-term trends in awareness, treatment, and control across HICs [9]. The comparative data provided quantitative benchmarks to contrast with country case studies, highlighting where HEARTS adopters or precursors have achieved faster gains compared with HICs that have stagnated. These findings document ongoing inequities and declining control levels and serve as a baseline against which to evaluate HEARTS implementation. Countries classified by the World Bank as low- or middle-income, such as Cuba and Saint Lucia, were excluded from the analysis despite their HEARTS experiences being included in PAHO documents [17].

### 2.3 Analytical approach

Consistent with the structured program report design and with the analytical approach of formal meta-analytic pooling was not performed, given the heterogeneity of data sources, variation in reporting periods, and differences in BP control threshold definitions across settings. Instead, the following analytical steps were applied:

Descriptive program characterization: Implementation characteristics were extracted from each source (year of adoption, number of facilities enrolled, catchment population size, scope of implementation, and available outcome data) and summarized in tabular form (Table 1, Table 2, Table 3, Table 4, Table 5).

**Table 1:** HEARTS Facilitators, Barriers, and Examples in High-Income Countries**.**

HEARTS Component [8]	Facilitators in HICs	Barriers in HICs	HEARTS Implementation Strategies	Measured Outcomes
**H: Healthy Lifestyle Counseling**	● Established public health campaigns (salt reduction, tobacco control, obesity prevention) [1]) ● Digital health tools widely available [7]	● Obesity epidemic ● Aging populations ● Processed food environments [5] ● Socioeconomic inequities in diet and exercise adherence [6]	● Structured lifestyle counseling integrated into PHC ● In Trinidad and Tobago, district health visitors and nutritionists provide counseling on lifestyle modifications [23] ● Saint Lucia and Barbados integrate counseling into PHC visits with registry follow-up [24]	● Trinidad and Tobago, Saint Lucia, and Barbados demonstrated 5–10% increases in counseling coverage within PHC registries [24]
**E: Evidence-Based Protocols**	● Established guidelines [5] ● Strong trial evidence (SPRINT) [9] ● Canadian CHEP and Kaiser models informed HEARTS design [10]	● Overlapping guidelines (ACC/AHA vs. ESC/ESH) [9] ● Therapeutic inertia [16] ● Polypharmacy challenges [5]	● HEARTS protocols standardize and simplify treatment algorithms	● Canada (CHEP): 13% → 66% control rates [11] ● U.S. Kaiser: 44% → 90% control rates [10] ● Barbados precursor program: 14.5 percentage point increase over 18 months in hypertension control ● Bermuda registry: control nearly doubled from 19% → 36% [12]
**A: Access to Essential Medicines and Technology**	● Broad insurance coverage- >90% of communities have access to antihypertensives [7] ● Validated devices available [8] ● Home BP monitoring common [1]	● Potentially high drug costs ● Medication adherence issues [5] ● Uninsured populations remain vulnerable [6]	● HEARTS ensures reliable drug and device access- U.S. telehealth refill systems [7] ● Japan home BP monitoring [1] ● Bermuda registry-driven therapy intensification [20] ● Trinidad and Tobago treatment algorithm designed for local medication access and reduced pill burden [23]	● The Bahamas hypertension pathway improved control with telmisartan, amlodipine, and chlorthalidone [19]
**R: Risk-Based CVD Management**	● Advanced laboratory and diagnostic capacity [5] ● CVD/CKD/diabetes calculators common [1] ● Aligns with HEARTS 2.0 integration [17]	● Multimorbidity increases complexity [1] ● Fragmented systems prevent integration [6]	● HEARTS integrates hypertension with broader CVD risk management and uses risk charts validated in HICs	● WHO HEARTS risk charts validated in Japan, South Korea, Singapore, Australia, and New Zealand [22]
**T: Team-Based Care**	● Multidisciplinary teams widely utilized [9] ● Telemedicine supports follow-up [7]	● Uneven workforce distribution [6] ● Scope-of-practice and payment constraints [16]	● HEARTS formalizes team-based PHC delivery	● U.S./Canada pharmacist-led programs: +15–20% control improvement ● Bermuda/Bahamas PHC teams [19,20] improved treatment coverage ● Chile expanded scope for nurses and pharmacists to titrate antihypertensives [25]
**S: Systems for Monitoring**	● Advanced EHRs [9] ● National surveys and registries [11] ● Physician quality metrics [11]	● Control stagnant since mid-2000s (<70% at best) [9]) ● Fragmented datasets across insurers and providers [6]	● HEARTS strengthens registries and monitoring with physician performance feedback	● Barbados registry: 10% → 12% (2022–2023) ● Trinidad and Tobago registry: 31% → 33% since 2019- Saint Lucia registry: 19% → 21% since 2023 [24] ● Bermuda registry: 25% → 62% (2023–2024) [20] ● Non-HEARTS countries (Germany/South Korea): unchanged; Finland/Spain: <50% [9]

**Table 2:** Implementation of HEARTS and Related Hypertension Programs in High-Income Countries.

Country	Year of Implementation/Program Start	Health Facilities Involved	Population Coverage	Scope of Implementation	Key Outcomes
Canada [11]	1999 (CHEP program, HEARTS inspiration)	National program across primary care	• National population, approximately 30 million in 1999	• National guideline and registry-based program	• Control improved 13% (1980s) → 66% (2006) [11] • Plateau and declines after 2011 when program priorities shifted
United States [10]	Early 2000s (Kaiser Permanente Northern California, HEARTS inspiration)	Kaiser Permanente integrated system	• ∼3.5 million adults in Northern California	• Registry-based program	• BP control improved <50% (2001) → >80% (2013) → 90% [26]
Barbados [21,24]	2022	8 PHC centers by 2020	• Catchment: 121,000 adults • Enrolled: 12,200 in hypertension registry	• First HEARTS pilot country • Transitioned to national rollout with registry-based monitoring	• Coverage increased from 10% in 2018 to 12% in 2020
Chile [27]	2018	National PHC centers	• Approximately 77% of national population (over 14 million in 2019)	• National PHC facilities • Expanded to nurses and pharmacists in 2019	• Treatment levels achieved 57% stability compared to 39% in controls from 2017 to 2019 • 227 PHC centers in 2019 → 387 PHC centers by 2021 • Hypertension stability decreased to 46% for treatment groups and 26% for controls by December 2021 due to COVID-19 pandemic
Trinidad and Tobago [28]	2019	National PHC centers	• Catchment: 143,810 adults • Enrolled: 12,527 in hypertension registry	• National HEARTS clinical pathway adoption	• Coverage improved from 31% in 2019 to 33% in 2020 • 5 PHC centers at launch (4.9%) → 46 PHC centers by 2021 (45%) • BP levels decreased from 155/96 to 123/75 [23]
Saint Lucia [24]	2023	National PHC facilities	• Catchment: 6336 adults • Enrolled: 1406 in hypertension registry	• National HEARTS clinical pathway adoption	• Coverage improved from 19% in 2019 to 21% in 2020
British Virgin Islands [29]	∼2020	BVIHSA hospital, 13 PHCs	• 5000–10,000 treated for hypertension [30] (HEARTS registry coverage not reported)	• Adopted formal hypertension drug protocol	• Amlodipine 10 mg → Step 2: + Lisinopril 20 mg → Step 3: + Lisinopril 40 mg → Step 4: + Indapamide 1.5 mg [31]
Bahamas [19]	∼2019	Public PHC facilities in The Bahamas Ministry of Health	• National coverage (∼398,000)	• National HEARTS clinical pathway adoption	• Pathway appraisal score 31.5/34 (93%), aligned with international standards
Bermuda [12,13]	2023	Public PHC clinics Hamilton Health Centre registry	• Registry expanded from 26 patients (2023) → 85 (Early 2024) → 110 (Dec 2024) • Control improved from 25% (2022 baseline) → 36% (2024) → 62% (Dec 2024)	• National rollout of HEARTS across PHC	• Registry expansion led to BP control nearly doubling to 36% at Hamilton Health Centre

Note: All BP control rates reflect the WHO HEARTS threshold of <140/90 mmHg unless otherwise indicated. US ACC/AHA guidelines define control as <130/80 mmHg; where US data are cited at this threshold, this is noted explicitly.

**Table 3:** Comparison of WHO HEARTS, Canadian Hypertension Education Program (CHEP), and Kaiser Permanente Hypertension Control Model.

Features	WHO HEARTS [8]	Canadian Hypertension Education Program (CHEP)	Kaiser Permanente Northern California [26]
**Origin and context**	• 2016 WHO initiative to strengthen CVD prevention and control in LMICs	• Established in 1999 as an annual guideline update process to improve awareness, diagnosis, treatment, and control of hypertension across Canada [32]	• Launched in 2000 after internal task force review to improve BP control in a large integrated U.S. health system
**Core components**	• Six modules: Healthy lifestyle counseling, Evidence-based protocols, Access to essential medicines/technology, Risk-based CVD management, Team-based care, Systems for monitoring	• Annual evidence-based recommendations • Evidence review • Key messages for clinicians • Pocket guides • Sodium reduction targets • Automated BP monitoring • ACEi/ARB/CCB-based regimens [33]	• Hypertension registry • Evidence-based guidelines with simplified algorithm • Promotes single-pill combinations • Centralized performance metrics
**Implementation approach**	• Adapted to national PHC systems- Applicable in LMICs	• Knowledge dissemination through interactive online lectures, websites, and community hypertension specialists [33]	• Organizational integration through EHRs • Registry-driven QI cycles • Team-based rollouts across medical centers
**Team-based care**	• Task sharing between nurses, pharmacists, and community health workers	• Multidisciplinary task sharing ensures that recommendations reach all provider levels [32]	• Physician-led teams supported by pharmacists, nurses, and medical assistants who conduct BP visits
**Monitoring/data systems**	• Standardized, national-level monitoring and evaluation guidance	• Surveillance through national surveys, prescription and hospitalization data, and tracking awareness, treatment, and control [32]	• EHR-enabled registry • Physician and clinic level dashboards for quality improvement
**Medication and protocols**	• Diuretics • ACEi/ARB • Statins • Metformin • Simplified country-level protocols	• Recommends ACEi/ARB, CCB/ACEi combinations for high-risk • Sodium intake <1500–2300 mg/day • Adopted automated BP devices [33]	• Single-pill lisinopril–HCTZ • Standardized drug protocols
**Knowledge training**	• WHO technical modules • Risk charts • Adaptable country guides	• Annual update cycle • Evidence reviews from Cochrane [32] • Promoting awareness through lectures, CME, websites, and pamphlets [33]	• Guideline distribution through print, pocket cards, online libraries, videoconferences, and local trainings
**Impact**	• Implemented in 32 countries as of 2020 • Countries that adopted early have improved BP control • Program helped improve medicine supply chains and created stronger systems for tracking progress	• By late 2000s, Canada reached world-leading BP control around 65–70% • Reduced stroke/MI hospitalization and mortality [32]	• 44% → 90% hypertension control from 2001 to 2013 • 24% decrease in MI • 42% decrease in stroke mortality
**Unique aspects**	• Modular LMIC-focused design • Utilizes lab and non-lab risk charts	• Annual evidence-based update cycle • Emphasis on sodium reduction [32]	• Registry and EHR integration • Medical assistant follow-ups • Simplified algorithm that promotes single-pills

**Table 4:** Barriers to HEARTS Implementation in LMICs and HICs.

Area	LMIC Barriers	HIC Barriers	Shared Barriers
**Medications and Supply**	Frequent medication stock-outs Unreliable drug orders and supply chains High prices Potential for poor quality medications [34]	Variation in drugs and doses Registries and feedback loops not synthesized Lack of availability of new fixed-dose combinations [17]	Both require steady access to combination drugs and reliable, validated BP devices
**Treatment Protocols**	Physicians hesitant to use combination therapy Supply issues limit choices Step-based treatment plans not consistently followed [34]	Physicians resist standardized protocols Slow uptake of simplified algorithms High variation between practices [35]	Achieving physician acceptance and adherence to standardized protocols is challenging
**Workforce Roles**	Shortage of physicians Legal restrictions prevent nurses from prescribing Many patients bypass primary care for hospitals [34]	Team-based care underutilized Care remains physician-centered [24]	Both require stronger team-based care LMICs need task-sharing for access HICs need task-sharing for efficiency
**Lifestyle and Equity**	Difficult to integrate into busy clinics High out-of-pocket costs Weak community follow-up Limited staff for community programs [34]	Counseling underutilized Uninsured or low-income groups have control rates similar to LMICs [14] Patients struggle to sustain diet and exercise changes [36]	Patient adherence to lifestyle modifications is challenging
**Risk-Based Care**	Limited laboratory capacity at PHC Heavy provider burden delays treatment and increases dropout [34]	Risk tools exist but are complex and time-intensive for physicians [35]	Balancing simplified care with detailed risk assessments is challenging
**Information Systems**	Many clinics still use paper- Digital systems are weak or fragmented [34] Tracking patients and follow-up is difficult [35]	EHR systems have poor interoperability Reporting burden causes feedback fatigue [36]	Both require reliable systems for QI without increasing workload
**Financing and Leadership**	Low government prioritization Affordability of lifelong treatment is a concern Health systems are often fragmented [37]	Difficult to maintain program adherence Need for financing systems for vulnerable populations [17]	Strong political support and sustained funding are needed in all countries
**Health System Culture**	Weak incentives for providers Little accountability Rare performance tracking [35]	Physicians skeptical of strict guidelines and population-level targets [36]	Both require cultural change to support accountability and standardized treatment

**Table 5:** Strengths of HEARTS Implementation in LMICs and HICs.

Area	LMIC Strengths	HIC Strengths
**System Organization**	Single Ministry of Health controls most PHCs, allowing nationwide rollout and rapid protocol dissemination [24,34]	Centralized ministries have substantial funding [28] Large integrated systems can spread protocols across many clinics simultaneously [25] Already trained nurses and pharmacists who can manage blood pressure [27]
**Innovation**	Simplified algorithms Fixed-dose combinations simplify treatment and improve adherence [34]	Stronger infrastructure enables large-scale program development [25]
**External Support**	Direct support from PAHO or CDC provides training, tools, and funds, accelerating implementation [34,36]	Ability to fund programs without donor dependence Can utilize external technical advice to improve programs [22]
**Monitoring and Registries**	No pre-existing national hypertension tracking system, so new registries are designed to align with HEARTS from inception [24,36]	Pre-existing EHRs and national quality reports Data platforms can automatically link to clinics and track results [2]
**Medication and Technology**	HEARTS has helped provide essential drugs and blood pressure devices where they were previously unavailable [24,34]	Reliable supply chains, validated blood pressure monitoring devices, and extensive drug formularies [22]

Pre- vs. post-implementation comparison: Where registry data were available (Bermuda, Barbados, Kaiser Permanente, CHEP), absolute percentage-point changes in BP control rate and treatment coverage from baseline to the most recent available reporting period were calculated and reported. No tests of statistical significance were applied, as this is a program evaluation report rather than a controlled trial.

Longitudinal trend analysis: NCD-RisC pooled survey data were used to characterize long-term trends in hypertension awareness, treatment, and control across 12 HICs from the 1980s through the most recent available survey period, providing a national-level comparator against which HEARTS adopter outcomes can be contextualized.

Structured comparative analysis: A predefined feature matrix (Table 3) was used to compare WHO HEARTS with pre-HEARTS reference models (CHEP and Kaiser Permanente) across eight dimensions: origin and context, core components, implementation approach, team-based care model, monitoring and data systems, medication protocols, knowledge and training, and impact. This matrix structure mirrors the comparative framework used in the Moran et al. LMIC analysis.

Implementation fidelity assessment: Where HEARTS pathway appraisal data were available (Bahamas), checklist compliance scores were extracted and reported as indicators of adherence to HEARTS standards.

## 3 Results

Table 1 summarizes how the six components of the WHO HEARTS technical package apply in HIC contexts and outlines the main facilitators that HICs possess, the barriers that persist, and solutions proven by HEARTS or related global models. Multimorbidity, socioeconomic inequities, fragmented data systems, and stagnating control rates demonstrate that resources alone are insufficient without coordinated frameworks. Unless otherwise noted, all BP control rates in this report reflect the WHO HEARTS threshold of <140/90 mmHg. In the United States, where the ACC/AHA guideline threshold of <130/80 mmHg applies, the nationally reported control rate was 21% in 2021–2023 at this stricter definition. This threshold difference should be considered when comparing US data to HEARTS program outcomes reported in this report. The examples from Bermuda, the Bahamas, Canada, and the United States illustrate that HEARTS can be effectively implemented in high-resource settings, producing measurable gains in coverage, adherence, and BP control.

Table 2 shows how HEARTS has been adopted in HICs, building on earlier models in Canada and the United States and expanding across the Caribbean and Latin America. CHEP and the Kaiser Permanente registry in California demonstrated the power of standardized protocols and monitoring systems, with control rates climbing from under 20% to over 60–80%. Chile's early adoption in 2018 led to rapid expansion from 227 to 387 primary care centers by 2021, supported by regulatory changes enabling nurses and pharmacists to titrate antihypertensive medications [27]. In Trinidad and Tobago, HEARTS launched in 2019 with 5 pilot clinics and scaled to 46 by 2021, supported by a national HEARTS Oversight Committee but challenged by system fragmentation and provider resistance [28]. Barbados followed with registry-linked implementation across two polyclinics, achieving a 14.5 percentage point increase in BP control over 18 months [21]. In 2022, Bermuda became a formal member of HEARTS and initiated implementation in 2023 by convening a national technical working group and developing a standardized clinical pathway [20]. The Bahamas entered through the second HEARTS cohort, adopting its first national hypertension pathway with the HEARTS appraisal checklist in 2023–2024 [17,19].

Table 3 compares the different strategies developed for improving hypertension control. WHO HEARTS, launched by WHO in 2016, was designed for use in LMICs, with simplified treatment protocols, strengthened supply systems, and a focus on task-sharing among doctors, nurses, and community health workers. Canada's CHEP program relies on annual evidence-based updates and public health measures such as sodium reduction, while Kaiser Permanente in Northern California demonstrates how an integrated U.S. health system can combine registries and EHRs with streamlined guidelines and team-based follow-up to drive control rates close to 90%. While CHEP and Kaiser demonstrate success in high-resource settings, HEARTS stands out for its flexibility and adaptability, offering a 6-module framework that can be scaled across different health systems and applied globally.

Fig. 1 compares the main elements of CHEP, Kaiser Permanente's hypertension model, and WHO HEARTS, showing which components from the first two have been incorporated into the HEARTS package. CHEP monitored hypertension at a national level through surveys and administrative data, while Kaiser created a unified electronic registry and dashboards. HEARTS includes a registry module as well, but in many countries, it initially operates as a hybrid paper and electronic system. CHEP also introduced national sodium reduction campaigns, while Kaiser focused its lifestyle efforts within its own system. In contrast, HEARTS provides structured lifestyle counseling in clinics, though broad food policy measures are implemented by governments. Kaiser linked its registry to major cardiovascular events such as myocardial infarction and stroke, while CHEP reported those outcomes at a population level. HEARTS currently concentrates on BP control and other process measures rather than routinely capturing those events. This figure demonstrates that HEARTS has incorporated most of these approaches into a modular package that functions across settings, while a few features such as fully integrated real-time dashboards, nationwide policy campaigns, and routine tracking of major clinical outcomes remain outside its core components.

**Fig. 1:**
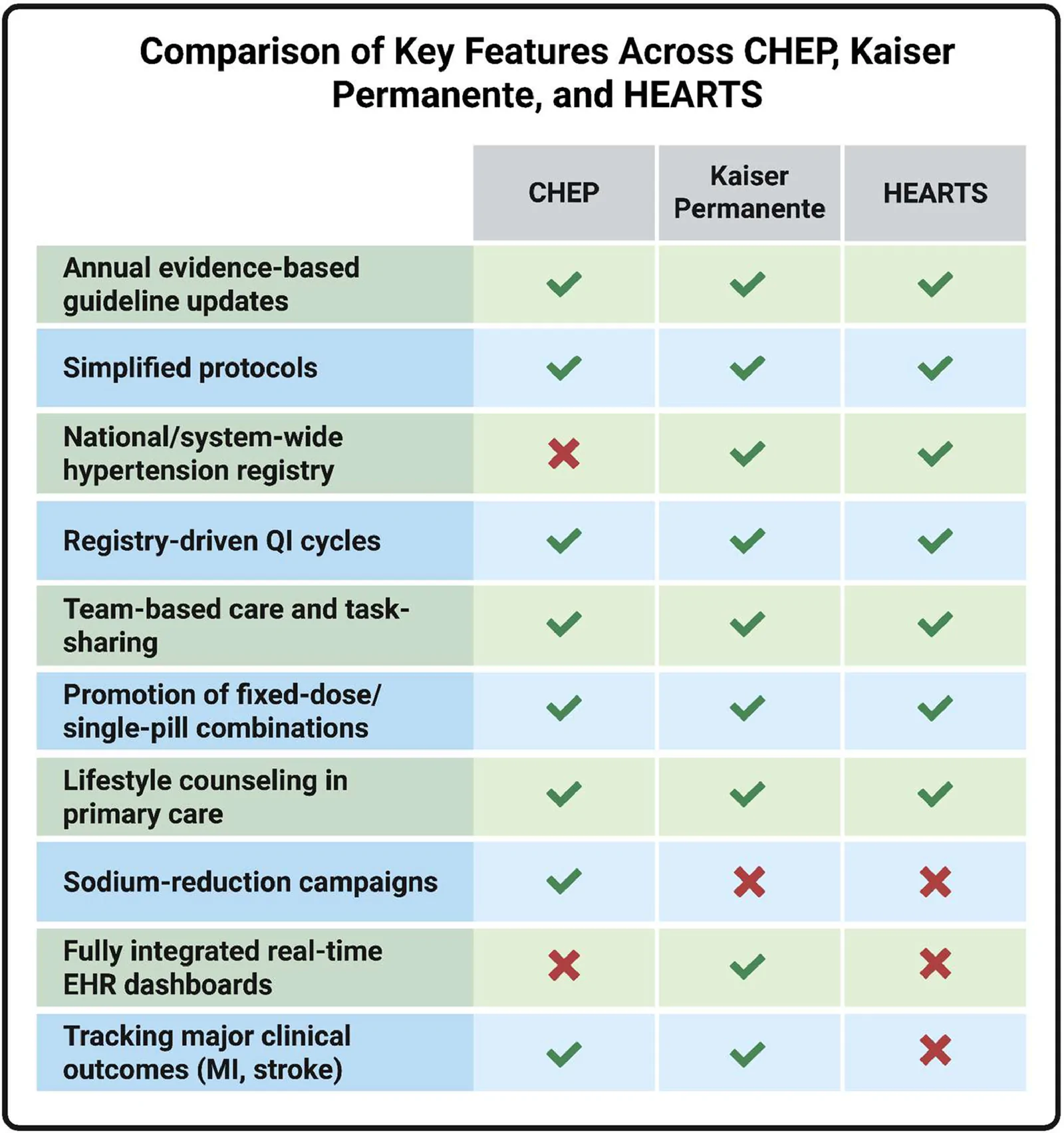
Comparison of key features across CHEP, Kaiser Permanente, and WHO HEARTS.

Fig. 2 summarizes hypertension control efforts across high-income countries by categorizing them into HEARTS adopters, precursor/model programs, and benchmark regions. On the map, HEARTS HIC adopters (green), HEARTS LMIC adopters (purple), models (blue), and benchmarks (orange) are highlighted. In the HEARTS HIC Adopters panel, Bermuda's HEARTS registry demonstrates rapid expansion with BP control surpassing 60% within two years, and the Bahamas' 2023 clinical pathway appraisal achieved 93% alignment with the HEARTS package. In the Precursor/Model panel, Barbados' early hypertension program demonstrated a 14.5 percentage point increase in control within 18 months, while Canada's CHEP program raised national control from 13% in the 1980s to 66% by 2006 before rates declined following a shift in program priorities. In the Benchmark Regions panel, Kaiser Permanente Northern California sustained control rates of 90%, the only program worldwide to exceed the global ceiling of 70%. This contrasts with U.S. national levels, which declined below 50% by 2020. Additional benchmarks in Germany, Finland, Spain, Japan, and South Korea plateaued between 45–65% and have never surpassed 70%.

**Fig. 2:**
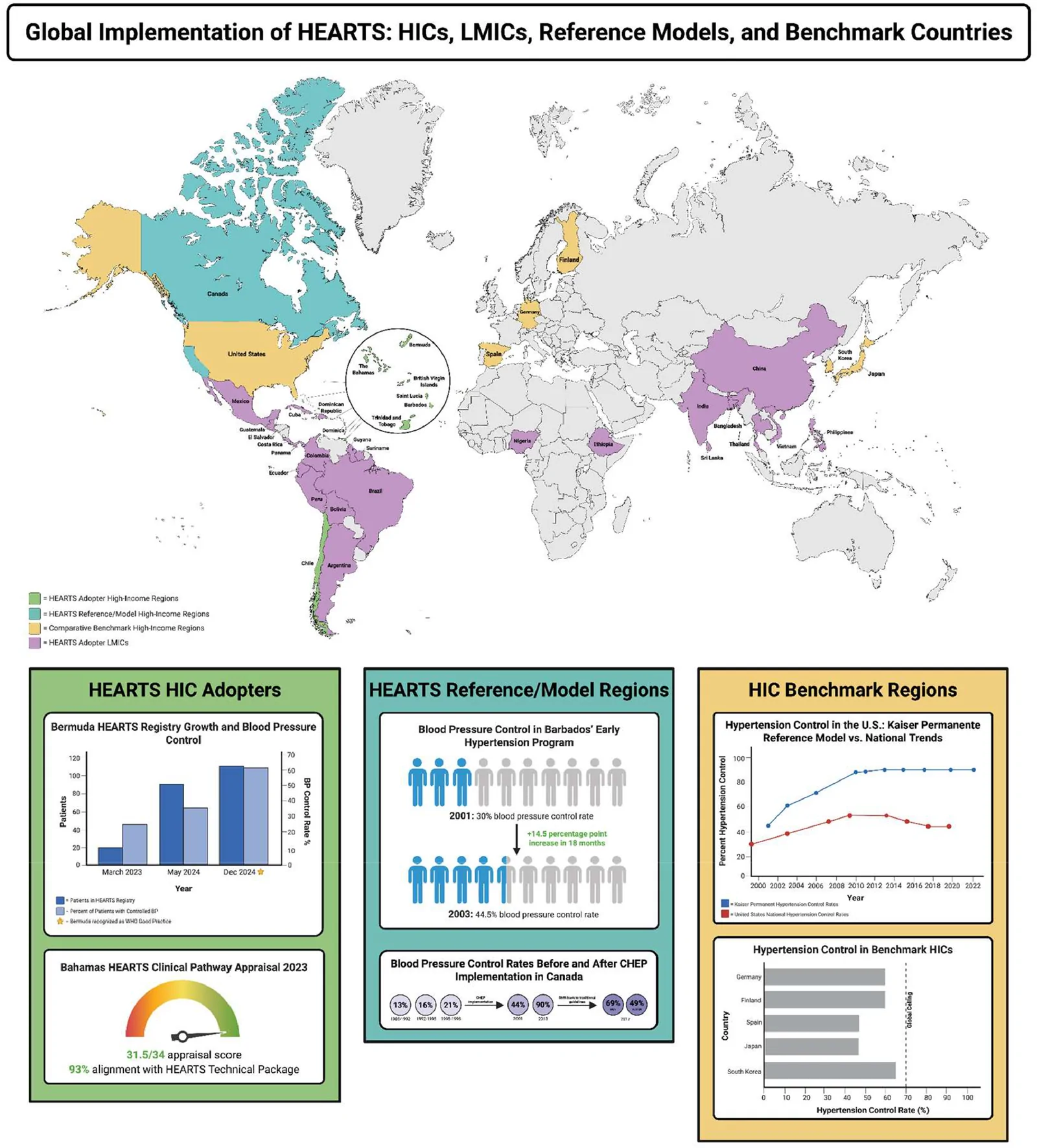
Global Implementation of HEARTS: HICs, LMICs, Reference Models, and Benchmark Countries.

These findings demonstrate that while robust infrastructure alone does not ensure progress, structured HEARTS-style frameworks and registry-based models consistently deliver sustained improvements in hypertension control. Compliance with HEARTS standards in HICs has been strong. In the Bahamas, adherence to the national clinical pathway was rated at 31.5 out of a possible 34 points, reflecting high compliance with HEARTS procedures from initiation [19]. Bermuda developed a pathway with risk-stratified targets and fixed-dose combinations that aligned with key drivers of HEARTS [12,20]. The HEARTS Compendium of Essential Clinical Tools and regional appraisal process facilitated these activities by providing standardized recommendations to ensure that HIC implementers adhered to key practices such as repeated BP measurement, simplified treatment regimens, task-sharing, and three-month medication refills [22]. These results indicate that HEARTS implementation can be rapidly initiated in health systems with adequate resource provision.

Table 4 demonstrates that barriers to implementing HEARTS differ by setting. In LMICs, many challenges stem from gaps in basic health system infrastructure. Medications frequently experience stock-outs, supply chains are unreliable, treatment protocols are not consistently followed, and typically only physicians are legally authorized to prescribe medication while insufficient numbers exist to meet demand. HICs are able to avoid many of the infrastructure shortages that impede HEARTS implementation in LMICs; however, substantial barriers remain. The most significant challenges are resistance to standardized treatment protocols, wide variation in practice across providers, fragmented digital systems, and physician-centered models of care. Both LMICs and HICs share barriers such as achieving consistent provider adherence to protocols, securing long-term political and financial commitment, and transforming health system culture to prioritize accountability and standardized care.

Table 5 illustrates the different structural facilitators in LMICs and HICs that benefit HEARTS implementation. LMICs can roll out protocols nationally, rapidly adopt fixed-dose medicines, and establish new registries to track patients with assistance from PAHO and CDC. HICs already possess robust infrastructure, trained nurses and pharmacists, reliable supply chains, and data systems that monitor results in real time. These strengths demonstrate that HEARTS can function across a range of resource and infrastructure settings, leveraging each country's existing advantages.

Fig. 3 illustrates how the number of people enrolled in HEARTS has grown in Barbados, Saint Lucia, and Trinidad and Tobago. The values for Barbados are derived directly from registry data, while the numbers for Saint Lucia and Trinidad and Tobago represent estimates based on known coverage percentages from PAHO [38]. Because HEARTS is designed to deliver standardized hypertension care and registry follow-up within routine primary care, patient enrollment provides a clear indicator of how widely these elements have been implemented. These combined reported and estimated values demonstrate that HEARTS is steadily extending its reach in all three countries, and the increase in enrollment reflects the program's growing impact on access to standardized hypertension care.

**Fig. 3:**
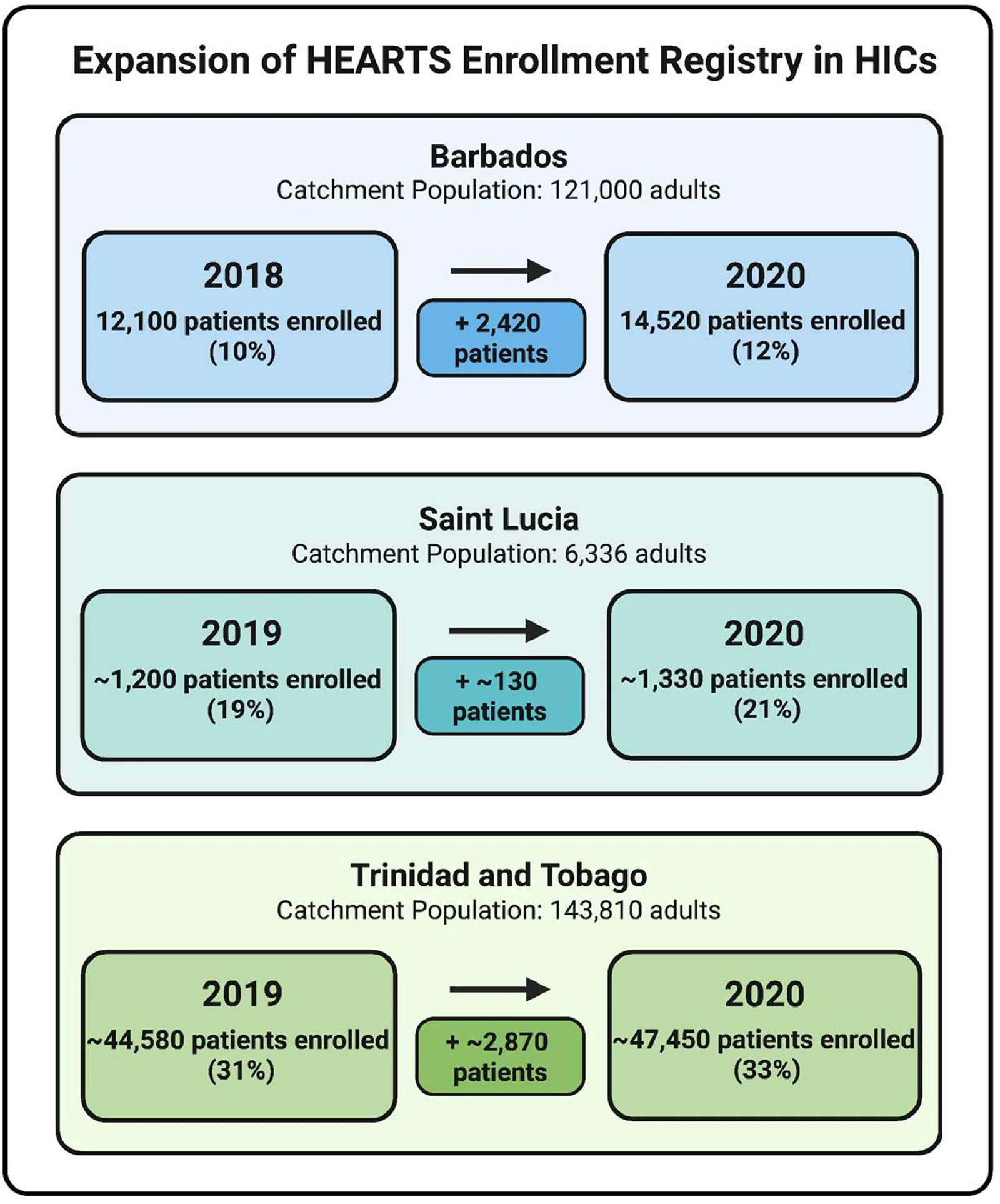
Expansion of HEARTS Enrollment Registry in HICs.

Early outcomes varied across these high-income adopters. In Chile through 2019, treatment and control rates remained stable at 57% and 39%, respectively, but declined during the COVID-19 pandemic to 46% and 26% [27]. Trinidad and Tobago's registry enrolled >12,500 patients across 46 facilities, with treatment coverage rising from 31% to 33% between 2019 and 2020, and patient testimonials documented substantial reductions in BP from 155/96 to 123/75 mmHg) following standardized care [23]. Saint Lucia reported more modest improvements, with coverage increasing from 19% to 21% over the same period. In Barbados, registry enrollment reached approximately 12,200 patients, with coverage improving from 10% in 2018 to 12% in 2020 [24]. In Bermuda, implementation has produced substantial improvements. The Hamilton Health Centre's hypertension registry expanded from 26 patients in 2023 to 85 patients in early 2024 and 110 by December 2024, and the proportion of patients with controlled BP rose from 25% at baseline to 62%. These improvements were achieved through policies that increased access to free medication for uninsured patients, task-sharing activities that enabled nurses to dispense and adjust therapy at the point of care, and regular Plan-Do-Check-Act quality cycles. In 2025, Bermuda was awarded recognition by PAHO/WHO as a Non-Communicable Disease Good Practice [13].

Fig. 4 demonstrates the rapid expansion of HEARTS across primary health care (PHC) facilities in Trinidad and Tobago and Chile, based on documented data from PAHO. Each new participating facility represents a clinic where standardized HEARTS protocols, validated BP devices, and team-based care are being implemented, and where hypertension care is reaching more patients. This facility-level growth is significant for HEARTS because it demonstrates that the program is becoming integrated into routine primary care. The rapid increase in participating centers reflects ministries of health reorganizing their hypertension service delivery models and demonstrates substantial momentum for bringing standardized hypertension care to HICs.

**Fig. 4:**
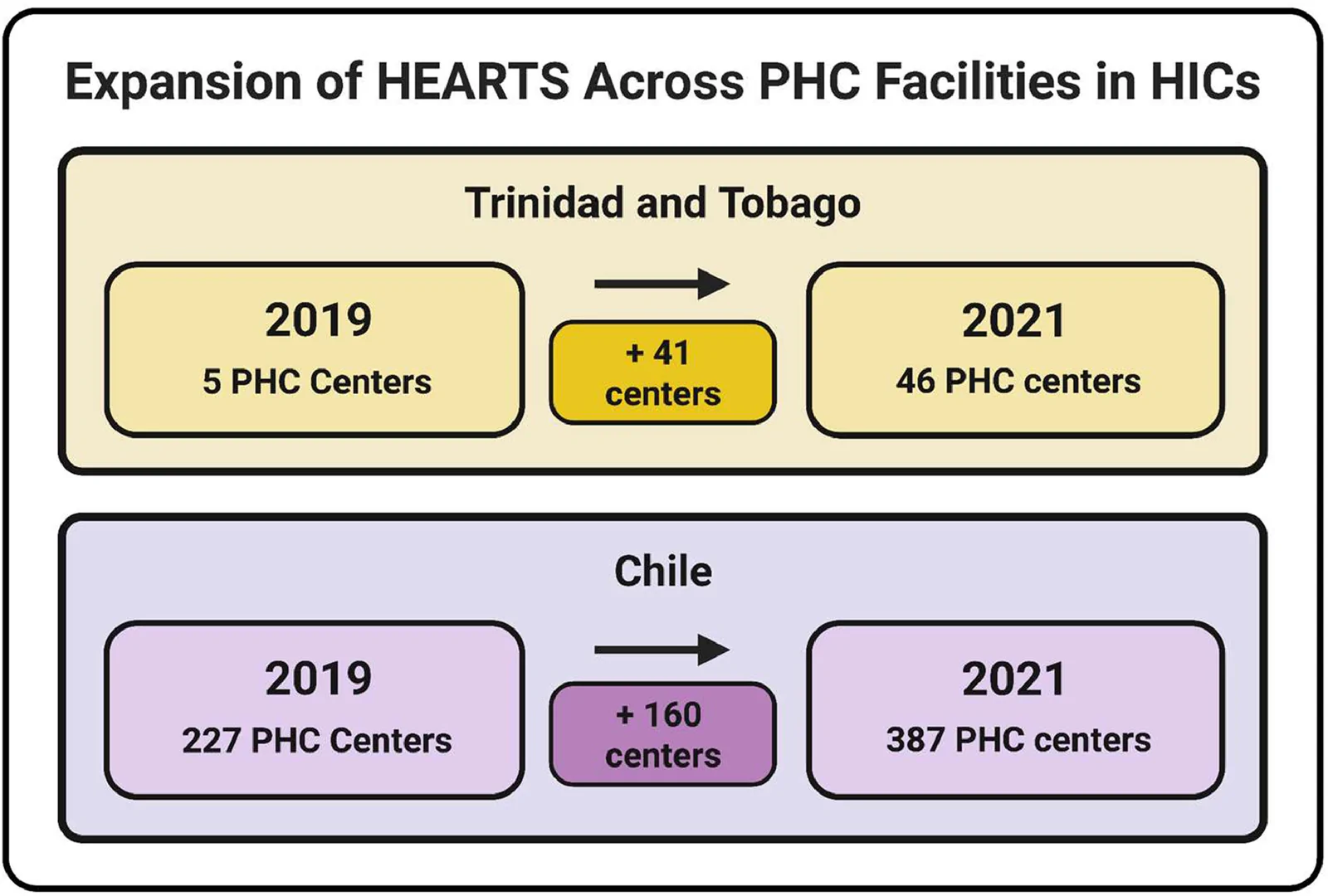
Expansion of HEARTS Across PHC Facilities in HICs.

## 4 Discussion

This report demonstrates that HEARTS can be effectively implemented in HICs, though it has been concentrated in LMICs as a cost-effective approach for noncommunicable disease management and was designed to address the most urgent gaps in lower-resource settings [39]. In many of these countries, primary care is fragmented, access to antihypertensive medications is limited, and data systems to track treatment are either weak or absent. Expansion across Latin America and the Caribbean has been made possible through technical support from PAHO and external funding from partners such as Resolve to Save Lives and the U.S. Centers for Disease Control and Prevention, both of which prioritize equity and vulnerable populations in their work. These factors explain why most early evaluations of HEARTS originate from LMICs, where the burden of uncontrolled hypertension is greatest.

However, the HEARTS initiative has proven feasible in high-income settings, and HICs face a different but equally pressing challenge. Hypertension control has plateaued or even declined in many wealthy settings, despite more advanced medical infrastructure. In the United States, control rates have dropped from approximately 50% to 44% in the past decade. Japan, Spain, and several European countries have reported similar stagnation. This demonstrates that resources alone are insufficient to guarantee effective BP management. Lack of standardized protocols, heavy reliance on individual physician judgment, and inadequate utilization of team-based care often impede progress.

Kaiser Permanente Northern California achieved BP control in >90% of patients by 2013 after introducing registry-based and team-driven care [10], while national U.S. control declined from nearly 60% in the mid-2010s to below 50% by 2020 [7]. This represents a subnational best practice within an integrated U.S. health system. However, at the national level, no high-income country has exceeded 70% hypertension control [9]. Canada's trajectory demonstrates how a HEARTS-type framework can drive improvement. Under CHEP, which emphasized standardized protocols, registry feedback, and continuous performance monitoring, national hypertension control climbed from 21% in the late 1990s to 66% by 2006, remaining near 70% through 2010 [9,11] Once program priorities shifted in 2011 away from these HEARTS-like features toward a more traditional guideline process, control rates stagnated and subsequently declined, with women falling from 69% to 49% by 2017 [9,11].

The cases of Bermuda, Barbados, Chile, Trinidad and Tobago, Saint Lucia, and the Bahamas suggest that HEARTS can provide substantial benefits in high-income contexts as well. Bermuda's control rates rose rapidly after adopting a registry-based approach. Barbados successfully integrated registry monitoring into its primary care network. Chile introduced regulatory reforms that enabled nurses and pharmacists to assume more active roles in hypertension management. In smaller island nations, HEARTS helped establish standardized protocols and monitoring systems, even in settings with limited administrative capacity. These examples demonstrate that HEARTS is not only workable in high-resource settings but also valuable as a mechanism to improve outcomes, reduce costs, and address equity gaps. Together, these experiences position HEARTS as more than a program for LMICs. They point to a universal model of cardiovascular risk management that is relevant across income levels. The global costs of hypertension are enormous, yet every dollar invested in control programs has been estimated to return eighteen dollars in economic benefit. Expanding HEARTS into HICs would help address declining control rates, reduce inequities, and shift care toward prevention.

The success of HEARTS in HICs demonstrates the relevance of structured system-level strategies even in well-resourced healthcare systems. The rapid improvements achieved in Bermuda were attributable to simplified protocols, fixed-dose combinations, equity-focused access to medicines, task-sharing with nurses, and Plan-Do-Check-Act quality cycles, all integral components of the HEARTS package. The Bahamas achieved high pathway appraisal scores on its first attempt, demonstrating how rapidly HEARTS can align national protocols with best practices at the country level. Barbados demonstrated over a decade ago that protocolized care and registries can enhance population-level control [21]. Collectively, these findings indicate that HEARTS can help HICs overcome obstacles such as therapeutic inertia, fragmented data systems, and persistent inequities. The adoption of HEARTS in HICs demonstrates that system-level strategies can lead to rapid improvement in hypertension control even in settings with strong baseline infrastructure. Bermuda's achievement reveals that registry-driven care, standardized protocols, and task-shifting can be scaled in a small yet high-income system and documented as best practice globally. Barbados and the Bahamas indicate that HEARTS offers a structured template for policy alignment, training, and monitoring. Meanwhile, stagnating control rates in larger HICs underscore the challenge of complacency and the importance of renewed investment in streamlined protocols and ongoing monitoring.

By contrast, among HEARTS adopters, Bermuda improved control from 25% at baseline to 62% within two years, while smaller island states such as Trinidad and Tobago, Saint Lucia, and Barbados documented incremental coverage gains through registry-based systems [24]. Additionally, Chile's decline during the COVID-19 pandemic highlights vulnerabilities to external crises, although the presence of standardized protocols facilitated monitoring and recovery planning [27]. The Canadian program further reinforces that HEARTS-style frameworks are not only effective but necessary to sustain performance. CHEP's registry-driven approach maintained national control among the highest globally, yet once its priorities shifted in 2011, rates declined sharply, especially among women [11]. Similarly, Kaiser Permanente's sustained 90% control demonstrates that registry utilization and standardized protocols, which are core HEARTS elements, are effective in HICs [10]. These contrasts highlight that universal insurance coverage or robust infrastructure alone do not guarantee high hypertension control, and that structured, protocol-driven approaches such as HEARTS are essential. Future implementations of HEARTS in the United States and other HICs with guideline thresholds below 140/90 mmHg will need to incorporate adapted BP targets and more intensive treatment algorithms than those currently embedded in the standard HEARTS package, representing an important adaptation challenge for high-income country scale-up.

LMICs also demonstrated how simplification can be advantageous. Stepwise treatment algorithms and fixed-dose combinations were adopted as cost-saving strategies, but they also reduced therapeutic inertia and improved adherence. HICs, despite having broader formularies and more advanced supply chains, continue to struggle with treatment intensification and physician-driven variation in prescribing. LMICs have outperformed HICs in this domain, demonstrating that streamlined protocols and task-sharing can achieve more consistent control [40].

Although HEARTS has proven beneficial in several HICs, significant barriers to comprehensive implementation remain. Many HICs already operate within complex health systems where hypertension management is influenced by multiple stakeholders, including insurers, hospital networks, and private providers [22]. This can limit the integration of standardized protocols and registries across the entire system [2]. By comparison, many LMICs deliver primary care through a single Ministry of Health, which enables them to introduce new protocols and data systems rapidly and uniformly [22]. Additionally, while there is greater availability of medicines and technologies, therapeutic inertia and reliance on physicians continue to delay treatment intensification. Even where evidence-based protocols exist, overburdened practices or professional restrictions may prevent broader adoption of task-sharing models with nurses and pharmacists. The limited uptake of simplified treatment algorithms and fixed-dose combinations also slows progress [[2], [41]]. By contrast, several LMIC implementations adopted simplified treatment plans and fixed-dose combination pills early, which reduced delays in care.

Inequities also persist within HICs, particularly among uninsured, low-income, or minority populations, who often face hypertension control rates comparable to those in LMICs [2]. Without equity-focused strategies, HEARTS implementation risks reinforcing rather than reducing these disparities [42]. Many LMIC pilots, while facing resource constraints, have been able to implement nationwide programs that reach more uniformly across their populations due to their centralized structures. Lastly, the robust technological infrastructure in HICs may actually present challenges, as EHRs, payer-based reporting systems, and regulatory environments are not always aligned with HEARTS' simplified, population-level model. In LMICs, registries were frequently built from the ground up and designed around HEARTS indicators from inception, avoiding some of the integration challenges observed in HICs. Overall, these limitations demonstrate that the obstacles to implementing HEARTS in HICs are shaped more by system complexity than by the fundamental gaps that drive implementation in LMICs [22].

The HEARTS initiative in HICs offers lessons for broader scaling of the program. Large healthcare systems in the United States, Europe, and Asia-Pacific would benefit from HEARTS' standardized protocols, registries, and ongoing monitoring. Integration with digital health platforms such as DHIS2 and mobile applications can further enhance monitoring and evaluation. Increased emphasis on multimorbidity and inequity in HICs is also required, given its elevated burden among older adults, low-income populations, and patients with diabetes or CKD [6]. Political commitment, financing for essential medicines, and institutionalization of team-based care are necessary to sustain improvements.

These priorities align with updated Phase 1 HEARTS 2.0 interventions released in May 2025, which recommend evidence-based strategies for integrating hypertension, diabetes, and CKD management, expanding non-physician providers' roles, and strengthening digital tools [17]. Expanding HEARTS across HICs can reverse stagnating hypertension control trends and result in long-term CVD reductions.

## 5 Conclusion

The implementation of HEARTS in HICs demonstrates that standardized, protocol-driven hypertension management frameworks are both feasible and effective across diverse healthcare contexts, regardless of resource level. The contrasting trajectories of HEARTS adopters versus benchmark HICs reveal a critical insight: robust infrastructure and financing, while necessary, are insufficient to achieve optimal hypertension control without coordinated system-level strategies that emphasize standardization, accountability, and continuous monitoring. Several key barriers to HEARTS implementation in HICs require attention: therapeutic inertia and physician resistance to standardized protocols; fragmented electronic health record systems that impede registry integration; persistent socioeconomic inequities in access and outcomes; and underutilization of non-physician providers despite available workforce capacity. However, HICs possess distinct advantages that facilitate implementation, including established monitoring infrastructure, trained multidisciplinary teams, reliable pharmaceutical supply chains, and financing mechanisms to support program sustainability. Expanding HEARTS across high-resource settings represents a critical opportunity to reverse declining control trends, reduce persistent inequities, and prevent avoidable cardiovascular disease globally. Future research should evaluate long-term sustainability, cost-effectiveness, and optimal strategies for integrating HEARTS with existing health systems in HICs.

## Data availability statement

All data are incorporated into the article and its online supplementary material.

## Funding

None.

## CRediT authorship contribution statement

**Jiancheng Ye:** Writing – review & editing, Writing – original draft, Visualization, Validation, Supervision, Software, Resources, Project administration, Methodology, Investigation, Funding acquisition, Formal analysis, Data curation, Conceptualization. **Sophie Bronstein:** Writing – review & editing, Writing – original draft. **Haoxin Chen:** Writing – review & editing, Writing – original draft.

## Declaration of competing interest

The authors declare that they have no known competing financial interests or personal relationships that could have appeared to influence the work reported in this paper.
